# The Multifold Etiologies of Limbal Stem Cell Deficiency: A Comprehensive Review on the Etiologies and Additional Treatment Options for Limbal Stem Cell Deficiency

**DOI:** 10.3390/jcm12134418

**Published:** 2023-06-30

**Authors:** Majid Moshirfar, Maliha Masud, Devon Hori Harvey, Carter Payne, Elayna Bruce, Yasmyne C. Ronquillo, Philip C. Hoopes

**Affiliations:** 1 Hoopes Vision Research Center, Hoopes Vision, Draper, UT 84020, USA; cpayne@hoopesvision.com (C.P.); yronquillo@hoopesvision.com (Y.C.R.); pch@hoopesvision.com (P.C.H.); 2 John A. Moran Eye Center, School of Medicine, University of Utah, Salt Lake City, UT 84132, USA; 3 Utah Lions Eye Bank, Murray, UT 84107, USA; 4 School of Medicine, University of Nevada Reno, Reno, NV 89557, USA; mmasud@med.unr.edu; 5 College of Medicine, The Ohio State University, Columbus, OH 43210, USA; harvey.605@osu.edu; 6 McGovern Medical School, University of Texas Health Science Center at Houston, Houston, TX 77030, USA; elayna.g.bruce@uth.tmc.edu

**Keywords:** limbal stem cells, limbal stem cell deficiency, Palisades of Vogt, aniridia, xeroderma pigmentosum, dry eye syndrome, meibomian gland dysfunction, contact lens-induced LSCD, ocular burn-induced LSCD, limbal stem cell transplantation

## Abstract

Given the various ocular manifestations of limbal stem cell insufficiency, an awareness of the genetic, acquired, and immunological causes and associated additional treatments of limbal stem cell deficiency (LSCD) is essential for providers. We performed a comprehensive review of the literature on the various etiologies and specific therapies for LSCD. The resources utilized in this review included Medline (PubMed), Embase, and Google Scholar. All English-language articles and case reports published from November 1986 through to October 2022 were reviewed in this study. There were collectively 99 articles on these topics. No other exclusion criteria were applied. Depending on the etiology, ocular manifestations of limbal stem cell deficiency range from dry eye syndrome and redness to more severe outcomes, including corneal ulceration, ocular surface failure, and vision loss. Identifying the source of damage for LSCD is critical in the treatment process, given that therapy may extend beyond the scope of the standard protocol, including artificial tears, refractive surgery, and allogeneic stem cell transplants. This comprehensive review of the literature demonstrates the various genetic, acquired, and immunological causes of LSCD and the spectrum of supplemental therapies available.

## 1. Introduction

**Figure 1:**
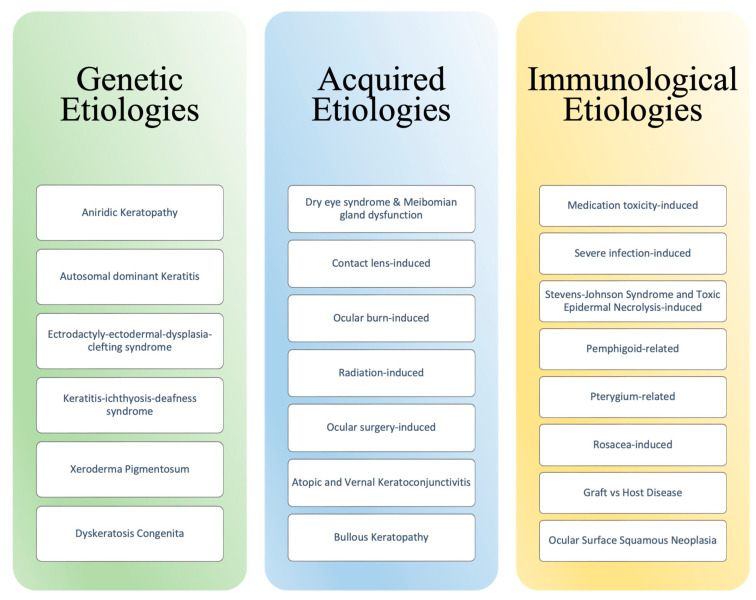
Etiologies of Limbal Stem Cell Deficiency.

Ocular homeostasis is maintained by several processes involving the eye’s structural layers, cell populations, and immunoregulatory responses. These processes involve the corneal stromal stem cells (CSSCs) and limbal epithelial stem cells (LESCs), each of which contributes to the regeneration of the corneal stromal layer and corneal surface, respectively [1]. Disruption to the corneal limbus, a well-defined layer of corneal stem cells between the sclera and cornea, often results in corneal epithelium irregularity and opacity, neovascularization, stromal scarring, and ulceration [2]. The likely etiology for this pathogenic process, termed limbal stem cell deficiency, includes a diverse array such as genetic, acquired, and immunologic. Etiologies of limbal stem cell deficiency have also been categorized as either LSC aplasia secondary to destruction or decreased function of LSC due to insufficient stromal support [3]. Figure 1 summarizes the many etiologies linked to LSCD. This comprehensive review of the literature discusses the diverse causes of limbal stem cell deficiency, the various diagnostic criteria, and the associated additional management options.

## 2. Limbal Stem Cells

The cornea can be separated into five distinct layers: corneal epithelial layer, Bowman’s membrane, corneal stroma layer, Descemet’s membrane, and corneal endothelium, from superficial to deep [4]. The limbus is a highly vascular and cellular-rich layer at the interface of the scleral and corneal layers. Within the limbus, rippling and folding of the basement membrane reveal crypts of the pluripotent stem cells, termed the Palisades of Vogt [5]. These limbal epithelial crypts are concentrated in the superior and inferior limbi and contain a high density of limbal epithelial stem cells [6]. LESCs function to maintain and restore the corneal surface following physical trauma or chemical insult [7]. The proposed mechanism for cellular turnover, known as the XYZ hypothesis, suggests that limbal stem cells proliferate and differentiate to give rise to cells that migrate centripetally along the basement membrane to the basal layers of the cornea before moving superficially to replace the epithelial cells that are shed [3,8,9]. The division and migration of limbal epithelial crypts to the cornea form a barrier to prevent encroachment of the conjunctival epithelium, maintaining the avascular and transparent conditions vital to corneal homeostasis. Corneal stromal-derived mesenchymal stem cells (CS-MSCs) inhabit the basal layer of the corneal epithelium and promote stromal support via extracellular matrix deposition, the expression of anti-apoptotic transcription factors, and the reinforcement of reconstruction and corneal repair of the limbus [4]. These mesenchymal stem cells can differentiate into keratinocytes and are found adjacent to LSCs within the stromal layer [4]. Studies have shown their corneal protective actions and proximity to LSCs significantly influence the health of the LSC microenvironment. Thus, disruption of the limbus and subsequent stromal support via injury to the CS-MSCs impairs the repair and reconstruction of the cornea [4]. Following disruption to the delicate stromal layer, the corneal epithelium is displaced by conjunctival epithelial cells (conjunctivalization), leading to neovascularization, corneal opacity, and subsequent loss in visual acuity [2].

## 3. Pathophysiology

A deficiency of limbal epithelial stem cells occurs in two ways: first, as limbal stem cell aplasia secondary to destruction via chemical burns, contact lens use, Stevens–Johnson syndrome (SJS), microbial keratitis, multiple surgeries or procedures; and second, insufficient stromal support, or an “abnormal microenvironment”, causing the decreased function of the limbal epithelial crypts [10]. The latter is seen in conditions such as aniridia, peripheral inflammatory disorder, chronic limbitis, or neurotrophic keratopathy [3]. Classification of LSCD falls under partial and complete deficiency, depending on the amount of residual LSCs present in the stromal layer and the degree of conjunctivalization of the corneal surface. Partial LSCD is defined by the presence of residual LSCs within the stromal layer, maintaining little stromal function and partial conjunctivalization of the corneal surface. In contrast, complete LSCD is characterized by a complete lack of LSCs in the limbus and the complete conjunctivalization of the corneal surface [11]. The most reliable indicator of limbal stem cell deficiency, conjunctivalization, refers to the migration or overgrowth of the conjunctival epithelial and goblet cells on the corneal surface, resulting in opacification and vision loss [3,9]. Compromise of the avascular state of the cornea occurs with neovascularization, where the balance between pro-angiogenic and anti-angiogenic factors is disrupted, resulting in a shift towards a pro-angiogenic state [12]. Recurrent corneal erosions, ulcers, or perforation of the cornea may also be seen [2,13].

## 4. Incidence and Prevalence

The current literature on LSCD shows that the leading cause is ocular surface burns [14]. Global trends for LSCD show that unilateral LSCD is more common than bilateral LSCD, with the most common causes being ocular surface burns for unilateral LSCD, while allergic conjunctivitis, SJS, toxic epidermal necrolysis (TEN), aniridia, and mucous membrane pemphigoid are seen for bilateral LSCD [14]. Provided the diversity in etiology for unilateral and bilateral LSCD, specialized approaches to treatment are required [14]. Gender-specific prevalence for LSCD is not definitive, considering a lack of agreement on the disease’s definition and diagnostic criteria; however, a higher prevalence of disease in young males is documented, with a majority suffering from total LSCD (male 2:1). Significant male predominance for chemical and thermal causes and a female predominance for contact lens-associated LSCD are also reported in the literature [14]. Age-related prevalence of LSCD demonstrates that patients presenting with the disease are, on average, middle-aged (42.9 years) and range from 24 to 43 years old, without sex predominance [15].

## 5. Clinical Presentation

Presentation of LSCD differs according to the etiology, and symptoms are often due to poor epithelial healing, resulting in decreased vision, pain from epithelial breakdown, foreign body sensation, conjunctival redness, and tearing [2,16]. Early symptoms of LSCD include irregular corneal epithelium and changes to or loss of Palisades of Vogt [16]. Depending on the degree of the limbus and LSC destruction, termed partial and complete, patients may present asymptomatically (in the case of partial LSCD) or with severe damage to the entire corneal surface (complete), resulting in functional blindness [16]. Awareness of the following clinical signs of a possible LSCD diagnosis includes symptoms secondary to reduced corneal epithelial repair and erosions, such as chronic conjunctival redness, foreign body sensation, photophobia, tearing, discomfort/pain, and decreased visual acuity [17,18]. This comprehensive review will discuss the different clinical presentations of LSCD.

## 6. Diagnosis and Prognosis

Diagnostic tools for LSCD include patient history, impression cytology for the presence of goblet cells on the cornea, in vivo laser scanning confocal microscopy (IVCM) of the limbus, anterior segment optical coherence tomography (AS-OCT) to measure the epithelial thickness and assess corneal vasculature, and direct histological staining (H&E and Papanicolaou staining) to assess the morphology of the epithelium [2,16]. The severity of LSCD is determined using a staging model based on the extent of the corneal and limbal involvement upon examination [19]. In the first stage, only the peripheral cornea is involved. Stage two involves the peripheral cornea in addition to the central 5 mm of the cornea, and in stage three, the entire cornea is affected. The ocular examination includes whether the visual axis, central 5 mm of the cornea, is involved (stages II and III) and whether greater than 50% of the LSC are intact [19]. Suitable treatment plans can be made, provided the diagnosis and staging are precise in determining the amount of residual LSCs remaining. Studies show that host LSCs had reconstructed injured corneal epithelium following allogeneic LSC transplantation [2]. However, no definitive prognosis exists for LSCD, given the different etiologies present [16].

## 7. Treatment Overview

Management of LSCD primarily follows a stepwise approach, focused on addressing the standard presentations seen in the disease and employing less invasive strategies first. Treatment starts with the discontinuation or limitation of the offending agent (e.g., contact lenses, medication, irritant exposures); next is the administration of corticosteroids for ocular surface inflammation, and thereafter, support to the residual limbal stem cells is offered via preservative-free lubricants and amniotic membrane transplants, and in severe cases, the restoration of stem cell reserves via a limbal stem cell transplant and penetrating keratoplasty is attempted [20]. LSC transplantations can be autologous from the fellow eye or allogenic from a donor. The graft may be directly transplanted in a single-stage procedure, or cells may be cultivated in a lab to be expanded and then transplanted at a later date in a two-stage procedure [21]. Furthermore, grafts may be obtained from various tissues, including a conjunctival limbal graft, keratolimbal graft, and simple limbal epithelial graft [21]. The specific details for which graft and surgery to implement will be indicated by the underlying etiology and stage of progression in each patient. Investigations on LSC transplantation and avenues of improvement are ongoing. Masood et al. described several therapeutic strategies to improve LSCD interventions, including the use of non-limbal stem cells to potentially restore LSC function. They described the potential to reconstitute mature corneal epithelial cells into LSC-like cells for transplantation via autologous cultivation [22]. An area of recent inquiry is the use of simple LSC transplantation vs. cultivated LSC transplantation. Both have been shown to have similar clinical efficacy [23]. However, Thokala et al. propose that simple LSC transplantation is superior and will be more common in the future compared to cultivated LSC transplantation due to the difficulties that accompany tissue cultures, including facilities, commercial cell-culture services, and the costs to maintain and expand cultures [24]. Despite the difficulties in cell cultivation, Jurkunas et al. have developed a novel and consistent manufacturing process for cultivated LSC transplantation, which may prove beneficial in the culture process [25]. In addition to the standard of care, therapies that specifically address the cause of the LSCD can be added to the management plan to further promote resolution. Etiologies and their additional therapies are discussed below.

## 8. Etiologies and Additional Treatments

### 8.1. Genetic Etiologies of LSCD

**Figure 2:**
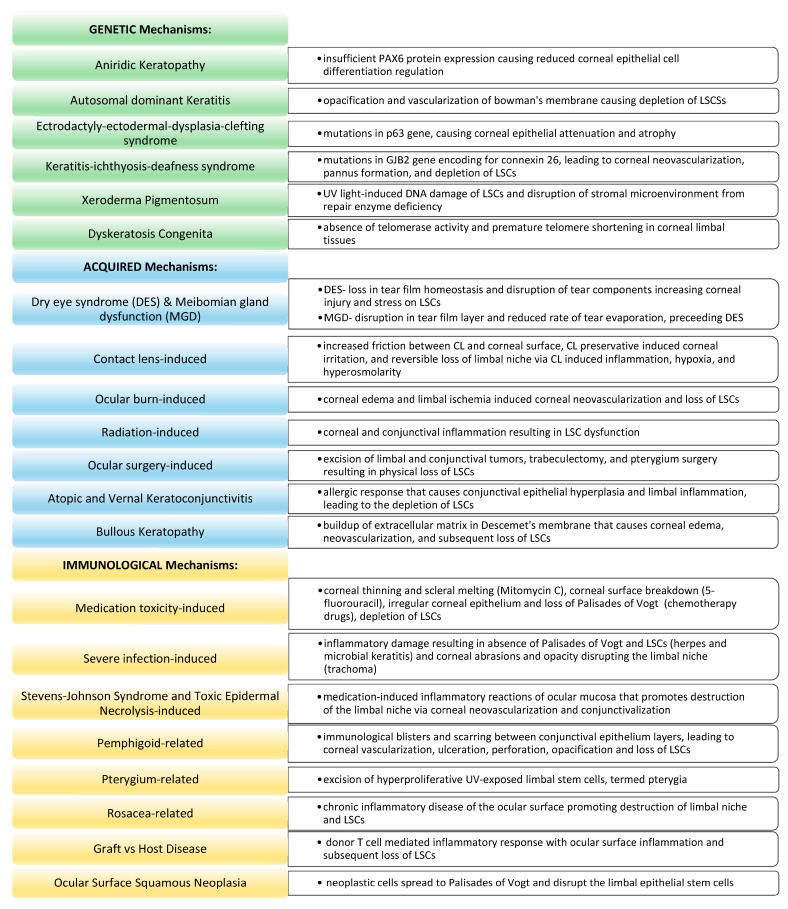
Mechanisms of Disease of Limbal Stem Cell Deficiency.

**Figure 3:**
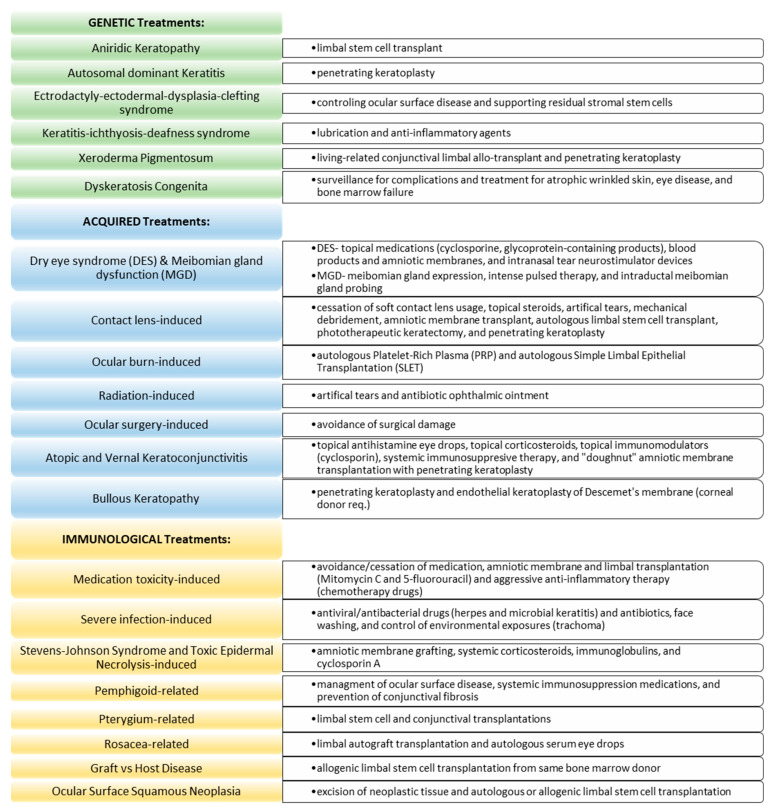
Specific Additional Treatment Options for Limbal Stem Cell Deficiency.

Genetic etiologies of LSCD include aniridia or aniridic keratopathy, ectrodactyly–ectodermal dysplasia–clefting syndrome (EEC), keratitis–ichthyosis–deafness (KID) syndrome, xeroderma pigmentosum, keratitis, and dyskeratosis congenita. The mechanism of injury and management associated with each genetic cause of LSCD is referenced in Figure 2 and Figure 3, respectively.

#### 8.1.1. Aniridic Keratopathy

LSCD from aniridic keratopathy is characterized by insufficient PAX6 protein expression, leading to severe congenital and corneal epithelial dysfunction and subsequent LSC deficiency. The PAX6 protein is a transcription factor expressed in embryonic ocular tissues and is involved in the regulation of corneal epithelial cell differentiation [26]. Evidence of aniridia keratopathy appears in the first decade of life, with symptoms ranging from decreased vision, foveal hypoplasia, nystagmus, amblyopia, and glaucoma [27]. A thickening and vascularization of the peripheral cornea, recurrent corneal erosions, ulcerations, and opacification result from reduced PAX6 gene expression [28]. In the early stages of the disease process, perseverative-free lubricants and amniotic membrane transplants help to support the residual limbal stem cells. In the case of severe LSCD by aniridic keratopathy, limbal stem cell transplants are the recommended course of intervention [28].

#### 8.1.2. Autosomal Dominant Keratitis

A variant of aniridia, hereditary keratitis is an autosomal-dominant disorder often diagnosed in childhood by recurrent stromal keratitis and vascularization of the anterior cornea [29]. It is a corneal degenerative ocular disease that develops early in life [29]. The pathogenesis of keratitis-induced LSCD involves the presence of a circumferential band of opacification and vascularization of the Bowman’s membrane adjacent to the stromal layer of the limbus, leading to the depletion of LSCs present therein [30]. Studies report that the addition of penetrating keratoplasty yields the most promising results in the treatment of the aniridia variant following compromised visual acuity [30].

#### 8.1.3. Ectrodactyly–Ectodermal Dysplasia–Clefting Syndrome

Ectrodactyly–ectodermal dysplasia–clefting syndrome (EEC) is an autosomal-dominant condition characterized by mutations in the p63 gene, belonging to a protein family transcriptionally responsible for the stem cell differentiation and embryogenesis in stratified epithelia [31]. The ocular defects involved in EEC include meibomian gland defects, reduced lacrimal gland secretion, evaporative dry eye, progressive corneal keratinocyte loss, and LSCD [32]. The pathogenesis behind EEC-induced LSCD concerns gene p63′s role in limbal cell migration, corneal differentiation, and corneal wound healing. Without p63 expression, corneal epithelial attenuation and atrophy are marked, leading to the development of LSCD [31]. Di Iorio et al. discuss that no such relationship exists between LSC failure and the severity of EEC and that LSCD is the major cause of visual morbidity in 60% of cases [31]. Management of EEC-induced LSCD is multimodal, considering the systemic effects of the disease on hair, skin, teeth, and sweat glands. Ocular treatment options include controlling ocular surface disease and supporting residual stromal stem cells via the standard methods of preservative-free lubricants and amniotic membrane transplants [33].

#### 8.1.4. Keratitis–Ichthyosis–Deafness Syndrome

Keratitis–ichthyosis–deafness (KID) syndrome is an autosomal-dominant condition resulting from mutations in the GJB2 gene encoding for connexin 26, a gap junction protein found in the epithelium of the inner ear and cornea [34]. KID syndrome is often diagnosed by the presence of sensorineural hearing loss, vascularizing keratitis, and skin manifestations, termed ichthyosis [34]. LSCD is a major pathologic outcome in KID by the corneal manifestations of vascularizing keratitis, pannus formation, and corneal neovascularization in the literature, all of which lead to the depletion of LSCs [35]. Management of the ocular surface manifestations of KID syndrome includes lubrication and anti-inflammatory agents [34].

#### 8.1.5. Xeroderma Pigmentosum

Xeroderma pigmentosum is an autosomal recessive condition characterized by cutaneous pigmentary abnormalities and neurological and systemic manifestations. Ocular defects in this condition include neovascularization, keratitis, and ocular surface neoplasia [31,36]. Common clinical presentations include photophobia, dry eyes, severe keratitis, pigmentation and atrophy of the lids, loss of lashes, and ocular surface neoplasms [37,38]. The source of LSCD in patients stems from a deficiency in the enzyme responsible for UV light-induced DNA damage repair, resulting in LSC exposure to UV radiation and a disruption of the stromal microenvironment [31]. Additional management for UV radiation-induced LSCD is currently a living-related conjunctival limbal allogenic transplant followed by penetrating keratoplasty. Avoidance and elimination of UV exposure in frequently visited environments are also recommended to prevent further damage [37].

#### 8.1.6. Dyskeratosis Congenita

The genetic etiology of LSCD, dyskeratosis congenita, also known as Zinsser–Cole–Engman syndrome, is a rare hereditary disease distinguished by a triad of reticulate hyperpigmentation, nail dystrophy, and leukoplakia [39]. In dyskeratosis congenita, mutations in 19 genes are linked to an absence of telomerase activity and premature telomere shortening [40]. Chen et al. discuss positive telomerase activity within the corneal limbal tissues, indicating the regenerative capability of the cells found within [41]. It is postulated that the absence of telomerase activity in dyskeratosis congenita results in the formation of LSCD and may be used as a biomarker for its diagnosis [41]. Additional treatment options are broad and concern the systemic manifestations of the disease, including atrophic wrinkled skin, eye disease, and bone marrow failure, and require surveillance for possible complications [42].

### 8.2. Acquired Etiologies of LSCD

Acquired etiologies of LSCD include ocular surface disease, contact lens-induced injury or trauma-induced (ocular burns, radiation, ocular surgery), atopic and vernal keratoconjunctivitis, and bullous keratopathy. Figure 2 and Figure 3 summarize the mechanism of injury and management associated with each acquired cause of LSCD.

#### 8.2.1. Dry Eye Syndrome and Meibomian Gland Dysfunction

Ocular surface diseases, dry eye syndrome (DES), and meibomian gland dysfunction (MGD) are major causes of LSCD, given their effects on ocular surface health. DES is a loss in tear film homeostasis following a disruption to the components of tears: mucin, lipids, or aqueous constituents, resulting in an ocular surface unfit to protect against environmental insult [43]. The clinical presentation of DES includes decreased tear production, irritation, and inflammation. Studies report damage to the central cornea and stressed LSCs in patients with DES, resulting in LSCD [43]. Santos et al. describe dry eye syndrome as the most important prognostic factor in corneal restorative procedures, such as conjunctival limbal grafts, concerning its effects on the ocular surface and the health of LSCs [44]. Advances in the supplementary treatments of DES-induced LSCD include topical medications such as cyclosporine or glycoprotein-containing products, blood products, and amniotic membranes to speed the healing of the cornea and decrease ocular surface inflammation, as well as intranasal tear neurostimulator devices to increase tear production [45]. Meibomian gland dysfunction (MGD), characterized by a disruption in the tear film layer, results in a reduced rate of tear evaporation, causing subsequent dry eye disease [46]. The clinical presentation and diagnosis of MGD overlap with DES and its implications in LSCD. Treatment of MGD comprises meibomian gland expression, intense pulsed therapy, and intraductal meibomian gland probing [43,47,48].

#### 8.2.2. Contact Lens-Induced LSCD

One of the most common but easily missed etiologies of LSCD is contact lens-induced LSCD (CL-LSCD). CL-LSCD is distinguished by its whorl-like epitheliopathy (opaque) extending from the superior limbus of the cornea and neovascularization, frequently diagnosed with a fluorescein stain and cobalt blue filter [17,49]. The clinical presentation for CL-LSCD in some patients is asymptomatic but can include blurred vision, eye pain, hyperemia, corneal conjunctivalization, and decreased visual acuity [17]. The proposed pathogenesis of CL-LSCD is three-part: first, due to a disruption in the tear film, there is a loss of lubrication and increased friction between the CL and the surface; secondly, harmful preservatives present in contact lenses irritate the corneal surface; lastly, CL-induced inflammation, hypoxia, and hyperosmolarity results in the reversible loss of normal limbal niche [50]. Deng et al. discuss the low oxygen permeability of CLs that result in an increased sensitivity of the corneal surface and the possibility to reverse LSCD if CL usage is decreased [51,52]. Corneal staining was also reported with silicone hydrogel lenses and multipurpose solutions resulting in irritation and damage to the ocular surface [53]. Conservative supplementary therapies for CL-LSCD include the termination of soft contact lens usage, reduction in the frequency of contact lens usage, topical steroids, and artificial tears [49]. Additional surgical and refractive treatments of CL-LSCD include mechanical debridement, amniotic membrane transplant, autologous limbal stem cell transplant, phototherapeutic keratectomy, and penetrating keratoplasty [50]. Termote et al. suggest that patients recovering from treatment utilize daily disposable contact lenses (if the patient insists on contacts) and avoid silicone hydrogel lenses and lens storage and cleaning solutions [49].

#### 8.2.3. Ocular Burn-Induced LSCD

Injury to the ocular surface via chemical or thermal burns, radiation, or ocular surgery is a serious and vision-threatening cause of LSCD. Chemical and thermal burns to the ocular surface, classified under chemical insults, are the leading cause of LSCD [54] and produce corneal edema and limbal ischemia, resulting in corneal neovascularization and conjunctivalization. One chemical noted to cause delayed LSCD is total body exposure to sulfur mustard [55]. Progression of a corneal burn leads to increased vascularization and disruption of the limbal layer, resulting in LSCD [54]. The severity of the burn to the ocular surface depends upon the degree of surface contact and penetration [56]. Dua et al. expanded upon the Roper–Hall classification on burn severity and the prognostic guidelines and described grade I injuries as little to no loss of LSCs, grade II as a subtotal loss of LSCs, while grade III refers to a complete loss of LSCs with residual conjunctival epithelium and vascularity in the proximal regions, and a grade IV injury wherein there is a complete loss of LSCs and proximal conjunctival epithelium [57]. Additional treatments for ocular burn-induced LSCD include autologous platelet-rich plasma (PRP), which prevents the progression of stromal melting, and autologous simple limbal epithelial transplantation (SLET) [23]. Moreover, it has been proposed that the anti-VEGF medication bevacizumab may play a role in the treatment of delayed LSCD in chemical insults by sulfur mustard [58].

#### 8.2.4. Radiation-Induced LSCD

Damage to the ocular surface from radiation therapy utilized in treating many systemic cancers has been documented to reduce the functioning of LSCs [59]. Fujishima et al. report on a case of corneal epithelial abnormality associated with conjunctival and corneal inflammation after radiation therapy for maxillary cancer in a 44-year-old male. Conjunctival epithelialization and goblet cells were identified in the superior and inferior areas of the cornea, resulting in stem cell dysfunction and loss of vision. The course of treatment, in this case, alongside standard therapies, included artificial tears and an antibiotic ophthalmic ointment resulting in the resolution of lost visual acuity and corneal abnormalities [59].

#### 8.2.5. Ocular Surgery-Induced LSCD

The destruction of the limbus and deficiency of limbal epithelial stem cells may be due to ocular surgical procedures, including the excision of limbal and conjunctival tumors, trabeculectomy, and pterygium surgery [19]. This form of surgically induced LSCD is termed iatrogenic and is confined to the sectors of the procedure.

#### 8.2.6. Atopic and Vernal Keratoconjunctivitis

Atopic and vernal keratoconjunctivitis are allergic conjunctival diseases characterized by ocular edema, thickening of the eyelid, corneal scarring and neovascularization, and tear film instability [60]. Atopic keratoconjunctivitis (AKC) is the most severe form of allergic conjunctival disease and is defined by bilateral atopic traits such as itchiness, dryness, redness, and blurred vision. Vernal keratoconjunctivitis (VKC) is a rarer and seasonal form of allergic conjunctivitis that presents with ocular pruritus, foreign body sensation, and photophobia [61]. VKC has been documented to include early age onset in teenagers, whereas AKC does not show an age-related differentiation and is more closely linked with asthma, rhinitis, and dermatitis [62]. AKC and VKC pathogeneses are mediated by inflammatory cells, such as T-helper cells and immunoglobulin E-mediated mast cells [60]. In VKC, the classification is based on the area of ocular involvement, including palpebral, limbal, and mixed (both palpebral and limbal) [62]. In AKC and VKC-induced LSCD, hyperplasia of the conjunctival epithelium, inflammatory cell infiltration, and limbal inflammation damage the limbal niche and progress to a loss of LSCs [60,61,62]. Supplemental management for AKC/VKC-induced LSCD includes topical antihistamine eye drops, topical corticosteroids, topical immunomodulators such as cyclosporin, and systemic immunosuppressive therapy [62,63]. Singh et al. also report on the promising management of partial LSCD in patients with AKC/VKC using a “doughnut” amniotic membrane transplantation with penetrating keratoplasty [64].

#### 8.2.7. Bullous Keratopathy

Bullous keratopathy (BK) is defined by a reduction in corneal endothelial cells (CEC), resulting in corneal thickening, haziness, and a subsequent loss of vision [61]. BK can be triggered by several corneal endothelial cell disorders, including Fuchs’s endothelial corneal dystrophy, wherein a progressive decline of CEC and the buildup of extracellular matrix in Descemet’s membrane results in corneal edema and a loss of visual acuity [65,66]. The clinical manifestations of LSCD in 16 patients with BK were the conjunctivalization of the peripheral cornea and delayed postoperative epithelialization in a study published in 2006 [67]. Other studies identified conjunctival goblet cells on the surface of the cornea and corneal neovascularization in patients with advanced cases of BK and suspected LSCD [68]. Additional management of BK-induced LSCD includes penetrating keratoplasty and endothelial keratoplasty of Descemet’s membrane, all of which require a corneal donor [65].

### 8.3. Immunologic Etiologies of LSCD

Immunological etiologies of LSCD include medication toxicity, severe infection (herpes, microbial keratitis, and trachoma), Stevens–Johnson syndrome (SJS), toxic epidermal necrolysis (TEN), mucous membrane pemphigoid, pterygium and pterygium excision, and rosacea. The mechanism of injury and management associated with each immunological cause of LSCD are summarized in Figure 2 and Figure 3, respectively.

#### 8.3.1. Medication Toxicity-Induced LSCD

Medication toxicity from Mitomycin C, 5-fluorouracil, and systemic chemotherapy with hydroxycarbamide have been proposed as causes of LSCD. Knowledge of these adverse reactions may be useful to ophthalmologists with patients at risk for limbal stem cell insufficiency in generating treatment plans. Sauder et al. discuss the link between LSCD and medication toxicity in an interventional case series following subconjunctival injections of Mitomycin C for the surgical treatment of glaucoma. The study documented corneal thinning and scleral melting in 43% of the patients following the injection, suggesting LSCD as a complication of subconjunctival Mitomycin C [69]. Similar associations have been demonstrated with the 5-fluorouracil application following glaucoma surgeries, wherein reduced vision and corneal surface breakdown were identified by impression cytology. Both partial and total LSCDs were confirmed in these cases involving 5-fluorouracil [70]. Finally, a few cases have been reported linking LSCD with the systemic chemotherapy drugs S-1 (an oral fluoropyrimidine derivative) and hydroxycarbamide. Histological examinations revealed a loss of the Palisades of Vogt at the superior limbus and irregular corneal epithelium in patients treated with S-1, as well as neovascularization of the peripheral cornea in patients treated with hydroxycarbamide [71,72]. Management of medication toxicity-induced LSCD follows a standard treatment protocol by avoidance/cessation of the medication, amniotic membrane transplantation, and limbal transplantation for Mitomycin C and 5-fluorouracil toxicity, and aggressive anti-inflammatory therapy in the case of anticancer drug toxicity [70,71,72].

#### 8.3.2. Severe Infection-Induced LSCD

Severe infection of the ocular surface and LSCD have been linked in numerous reports within the literature. Of those reports, herpes simplex keratitis and herpes zoster ophthalmicus, microbial keratitis, and trachoma were the most referenced infections to cause limbal stem cell insufficiency. Herpes simplex virus type 1 keratitis (HSK) is an infectious disease characterized by epithelial keratitis, which may progress to corneal opacification, corneal scarring, neovascularization, and loss of vision [73]. HSV’s counterpart, the herpes zoster ophthalmicus infection (HZO) from the varicella-zoster virus, is distinguished by its ocular manifestations, including conjunctivitis, uveitis, episcleritis, keratitis, and retinitis [74]. Liu et al. reported in their study from 2021 that patients with unilateral HSK and HZO demonstrated an absence of Palisades of Vogt following damage from inflammation and a significant loss in limbal stem cells [75]. Current supplemental management options include antiviral therapies to decrease the disease’s duration and severity [69]. Microbial keratitis is another infectious disease targeting ocular surface tissue via bacteria, fungi, and protist pathogens. In microbial keratitis, severe ocular surface inflammation and damage from the infectious agent promote necrosis of the limbal stem cells, resulting in LSCD [19]. The clinical presentation for microbial keratitis most commonly includes redness, pain, tearing, blurred vision, and inflammation [76]. Finally, trachoma, an infection of the conjunctiva by chlamydia trachomatis, results in corneal opacity, corneal abrasions from inverted eyelashes (trichiasis), scarring of the tarsal conjunctiva, and a possible loss of vision [77]. Trachoma-induced LSCD occurs following chronic microtrauma to the corneal surface from inverted eyelashes (trichiasis) and the subsequent disruption of the limbal niche [19]. Additional interventions for trachoma-induced LSCD are still being studied today but include antibiotics, face washing, and the control of environmental factors promoting the spread of the chlamydia trachomatis virus [77].

#### 8.3.3. Stevens–Johnson Syndrome and Toxic Epidermal Necrolysis-Induced LSC

Stevens–Johnson syndrome (SJS) and toxic epidermal necrolysis (TEN) are severe medication-induced inflammatory reactions of the skin and mucosa in the eyes, mouth, and genitals [78]. SJS and TEN manifest first with flu-like symptoms and progress to severe mucous membrane lesions in the eyes, mouth, and genitals [79]. The clinical presentation includes dry eye, lid-margin keratinization, corneal neovascularization, and eventually LSCD [80]. Ueta et al. discuss how multi-ingredient cold medications and non-steroidal anti-inflammatory drugs are the major eliciting drugs in patients with SJS/TEN and can be used as predictive factors alongside age [81]. Both inflammatory reactions can be classified under the acute and chronic stages according to what parts of the ocular surface are involved [78]. The severity of SJS/TEN can be determined by a grading system proposed by Sotozono et al., based on the presence of conjunctivitis, corneal epithelial defect, and pseudo membrane formation: 0 (zero) being no ocular involvement and 3, being the presence of both an ocular surface defect and pseudo membrane formation [81]. A positive correlation between acute systemic involvement and the development of LSCD was discussed in a retrospective case series by Choi et al., where corneal LSCD occurred in 32% of patients with SJS/TEN. Cytokines, such as interleukin (IL)-1B, matrix metalloproteinases, tumor necrosis factor-alpha, and vascular endothelial growth factor (VEGF), are increased in these inflammatory reactions and promote the destruction of the limbal niche via corneal neovascularization and conjunctivalization [82]. Additional management options for SJS/TEN-induced LSCD are difficult, considering that many cases report irreversible damage to the ocular surface [81]. However, in the case of acute SJS/TEN, amniotic membrane grafting, systemic corticosteroids, immunoglobulins, and cyclosporin A have been implicated as viable treatment options to further prevent further damage [79,80].

#### 8.3.4. Pemphigoid-Related LSCD

Ocular mucous membrane pemphigoid (OcMMP) is an immunological condition of the conjunctiva, wherein blisters form between the conjunctival epithelium and sub-epithelium and where significant scarring of the conjunctiva develops. OcMMP damages mucous membranes by IgA, IgG, and C3 deposition [19]. As the condition progresses, damage to the cornea manifests as corneal vascularization and opacification, ulceration, and perforation [83]. The clinical presentation depends on how quickly the conjunctivitis progresses, ranging from acute conjunctivitis and limbitis to ocular surface failure [84]. Regarding LSCD, OcMMP may include lacrimal duct scarring, which causes severe dry eye syndrome, and results in worsening LSC functioning [85]. Auxiliary treatment of OcMMP-induced LSCD includes the management of ocular surface disease, systemic immunosuppression medications, and the prevention of conjunctival fibrosis [84].

#### 8.3.5. Pterygium-Related LSCD

A pterygium is an ocular surface lesion postulated to derive from UV-exposed limbal stem cells. These lesions often invade tissues between the Bowman’s membrane and corneal epithelium and lead to epithelial proliferation, goblet cell hyperplasia, stromal plaques, inflammation, and Bowman’s membrane dissolution [86]. The pathology behind pterygium, as reported by Das et al., proposed that hyperproliferative epithelial cells within the limbal microenvironment cause the formation of pterygia within the corneal epithelium [87]. Thus, the removal of pterygia may induce LSCD and increase the ocular surface damage associated with the excision [19]. Proposed additional treatments mentioned in the literature include limbal stem cell and conjunctival transplantations [88].

#### 8.3.6. Rosacea-Induced LSCD

As discussed in the literature, physical injury to the stroma, as seen in ocular burns, SJS/TEN, OcMMP, contact lens wear, and severe infections to the ocular surface, results in the destruction of limbal stem cells and the disruption of the limbal niche, termed secondary LSCD. Ocular rosacea, a chronic inflammatory disease, is characterized by inflammation of the ocular surface, blepharitis, tear film instability, conjunctivitis and, in the most severe cases, corneal neovascularization and vision loss [89]. Corneal neovascularization, seen in patients with ocular rosacea, is commonly found growing from the superior limbus, suggesting damage to and the insufficiency of LSCs [89]. Supplemental treatment options for LSCD secondary to ocular rosacea, proposed in the literature, include limbal autograft transplantation or autologous serum eye drops to enhance corneal epithelialization [90].

#### 8.3.7. Graft vs. Host Disease

For patients with hematologic malignancies and diseases, a hematopoietic stem cell transplantation (HSCT) has curative potential. One of the major complications of a HSCT is graft vs. host disease (GVHD), which occurs due to a donor T-cell response against the host tissues, most commonly, minor histocompatibility antigens [91]. Ocular manifestations of GVHD include new-onset dry eye with inflammation of the ocular surface, which includes the cornea, conjunctiva, eyelids, meibomian glands, and lacrimal glands. This may lead to keratoconjunctivitis, cicatricial conjunctivitis, areas of punctate keratopathies or more severe complications, such as ulceration, perforation, or LSCD [92]. Allogenic LSC transplantation is the foremost treatment for bilateral LSCD, though it carries the risk of immunorejection [92]. In an effort to avoid rejection, two cases of LSCD secondary to GVHD were reported using a paired LSC and conjunctival transplant that was harvested from the same bone marrow donor. After successful LSC and conjunctival transplantation, immunosuppression was not indicated following one-year post-operation, at which time the grafts were stable [93]. Therefore, in cases of patients with LSCD secondary to GVHD, an allogenic LSC transplantation from the same bone marrow donor may be the most appropriate treatment.

#### 8.3.8. Ocular Surface Squamous Neoplasia

Ocular surface squamous neoplasia (OSSN) is a term that includes the following spectrum of conditions: conjunctival intraepithelial neoplasia, corneal intraepithelial neoplasia, and squamous cell carcinoma of the conjunctiva. OSSN is an extremely rare disease, with only 17–20 per million new diagnoses each year [94]. Symptoms may include minor to severe pain and changes in vision, including a total loss of vision [95]. The progression to carcinoma occurs when dysplastic epithelial cells obtain more malignant characteristics until they can invade through the basement membrane. The disease may originate from different locations within the corneal and conjunctival epithelium but is most commonly derived from the limbus [95], where a disruption of the Palisades of Vogt may occur [96]. While extremely rare, OSSN has been reported as a cause of LSCD [96,97,98,99]. For patients with OSSN-induced LSCD, additional management options focus on treating the underlying disease. Excision of the malignancy with clear margins is the preferred form of treatment for OSSN [95]. After neoplastic tissue excision, an autologous LSC transplantation is the most suitable for unilateral cases of LSCD secondary to OSSN; otherwise, an allogenic LSC transplantation is the most appropriate for bilateral LSCD secondary to bilateral OSSN. Bilateral OSSN has been reported alongside infections of human papillomavirus type 16 and xeroderma pigmentosa, although instances of this are rare [95].

## 9. Conclusions

Limbal epithelial stem cells are a complex component of the ocular surface, vulnerable to a multitude of cellular processes and environmental insults. Upon diagnosis of LSCD, the source of damage is key to providing the appropriate plan of treatment, given the nature of damage that each etiology ensues. Injury via genetic causes, such as aniridic keratopathy, keratitis, EEC, KID syndrome, xeroderma pigmentosum, and dyskeratosis congenita, convey the following treatments in addition to the standard protocol: limbal stem cell transplantation, penetrating keratoplasty, supportive care to residual LSCs and the ocular surface, lubrication and anti-inflammatory agents, and a UV exposure protocol, respectively. Injury via acquired causes, including DES and MGD, contact lens-induced, trauma-induced (ocular burns, radiation, ocular trauma), atopic and vernal keratoconjunctivitis, and bullous keratopathy indicate the following supplemental treatments: DES topical medications, MGD expression and probing, topical steroids and surgical/refractive procedures, autologous platelet-rich-plasma, antihistamine therapies, and penetrating/endothelial keratoplasty, respectively. Lastly, injury via the immunological processes include medication toxicity, severe infections, SJS/TEN, OcMMP, pterygium-induced, rosacea, GVHD-induced, and OSSN-associated LSCD suggest the following therapies in addition to the standard: amniotic membrane transplantation and anti-inflammatory therapy, antibiotics and antiviral medications, systemic corticosteroids and immunoglobulins, ocular surface disease therapies, limbal stem cell and conjunctival transplantations, autologous serum eye drops, allogenic LSC transplantations from the same bone marrow donor, and neoplastic excision with autologous/allogenic LSC transplantations, respectively.

## 10. Literature Search

The following resources were used to search the peer-reviewed literature: Medline (PubMed), Embase, and Google Scholar. The keywords used in the search included: limbal stem cells, LSCD, cornea, cornea stromal layer, genetics of limbal stem cells, aniridic keratopathy, keratitis, ectrodactyly-ectodermal-dysplasia-clefting syndrome, keratitis-ichthyosis-deafness syndrome, xeroderma pigmentosum, dyskeratosis congenita, dry eye syndrome, meibomian gland dysfunction, contact lens limbal deficiency, ocular burn limbal deficiency, radiation limbal stem cells, trabeculectomy, pterygium surgery, atopic/vernal keratoconjunctivitis, bullous keratopathy, mitomycin c limbal stem cell, herpes limbal stem cell, microbial keratitis, trachoma, Stevens-Johnson syndrome, toxic epidermal necrolysis, pemphigoid, rosacea, graft vs. host disease limbal stem cell, and squamous cell conjunctival carcinoma limbal stem cell. All English-language articles and case reports published from November 1986 through to October 2022 were reviewed in this study. There were collectively 93 articles on these topics.

## Data Availability

Databases used for review of the literature can be found at the following websites: https://pubmed.ncbi.nlm.nih.gov/, https://scholar.google.com/, and https://www.embase.com/.
